# Presence of Broad-Spectrum Beta-Lactamase-Producing *Enterobacteriaceae* in Zoo Mammals

**DOI:** 10.3390/microorganisms9040834

**Published:** 2021-04-14

**Authors:** Chloë De Witte, Nick Vereecke, Sebastiaan Theuns, Claudia De Ruyck, Francis Vercammen, Tim Bouts, Filip Boyen, Hans Nauwynck, Freddy Haesebrouck

**Affiliations:** 1 Department of Pathology, Bacteriology and Avian Diseases, Faculty of Veterinary Medicine, Ghent University, 9820 Merelbeke, Belgium; claudia.vermeir@gmail.com (C.D.R.); filip.boyen@ugent.be (F.B.); freddy.haesebrouck@ugent.be (F.H.); 2 Department Virology, Parasitology, Immunology, Faculty of Veterinary Medicine, Ghent University, 9820 Merelbeke, Belgium; nick.vereecke@ugent.be (N.V.); sebastiaan.theuns@ugent.be (S.T.); hans.nauwynck@ugent.be (H.N.); 3 Veterinarian/Zoo Health Management, Zoo Antwerpen, 2000 Antwerpen, Belgium; francis.vercammen@kmda.org; 4 Veterinarian/Zoo Health Management, Pairi Daiza, 7940 Brugelette, Belgium; Tim.Bouts@pairidaiza.eu

**Keywords:** *Escherichia coli*, zoo animals, antimicrobial resistance, zoonotic, BSBLs

## Abstract

Broad-spectrum beta-lactamase (BSBL)-producing *Enterobacteriaceae* impose public health threats. With increased popularity of zoos, exotic animals are brought in close proximity of humans, making them important BSBL reservoirs. However, not much is known on the presence of BSBLs in zoos in Western Europe. Fecal carriage of BSBL-producing *Enterobacteriaceae* was investigated in 38 zoo mammals from two Belgian zoos. Presence of *bla*-genes was investigated using PCR, followed by whole-genome sequencing and Fourier-transform infrared spectroscopy to cluster acquired resistance encoding genes and clonality of BSBL-producing isolates. Thirty-five putatively ceftiofur-resistant isolates were obtained from 52.6% of the zoo mammals. Most isolates were identified as *E. coli* (25/35), of which 64.0% showed multidrug resistance (MDR). Most frequently detected *bla*-genes were CTX-M-1 (17/25) and TEM-1 (4/25). Phylogenetic trees confirmed clustering of almost all *E. coli* isolates obtained from the same animal species. Clustering of five isolates from an Amur tiger, an Amur leopard, and a spectacled bear was observed in Zoo 1, as well as for five isolates from a spotted hyena and an African lion in Zoo 2. This might indicate clonal expansion of an *E. coli* strain in both zoos. In conclusion, MDR BSBL-producing bacteria were shown to be present in the fecal microbiota of zoo mammals in two zoos in Belgium. Further research is necessary to investigate if these bacteria pose zoonotic and health risks.

## 1. Introduction

The extensive use of β-lactams in human and veterinary medicine has favored global spread of broad-spectrum beta-lactamase (BSBL)-producing bacteria, especially in commensal *Enterobacteriaceae* [1]. These enzymes hydrolyze the amide bond of the β-lactam ring, rendering the antimicrobial ineffective [2]. The most widely used classification scheme for β-lactamases is the Ambler system, which divides β-lactamases into four classes based on their amino acid sequences (A to D). Class A (TEM, SHV and CTX-M enzymes), C (AmpC enzymes) and D (OXA enzymes) function by the serine ester hydrolysis mechanism, while class B (metallo-β-lactamase (MBL) enzymes) use a zinc ion to attack the β-lactam ring. All CTX-M-enzymes, most TEM- and SHV-enzymes and some OXA enzymes (i.e., OXA-10 and OXA-13 to OXA-19) are extended-spectrum β-lactamases (ESBLs). ESBLs hydrolyze most β-lactam substrates, except for cephamycins and carbapenems, and are inactivated by β-lactamase inhibitors. As TEM-1, TEM-2 and TEM-13 are only able to hydrolyze penicillins, and most OXA-type β-lactamases do not hydrolyze extended-spectrum cephalosporins, they are not considered ESBLs. On the other hand, AmpC hydrolyze all β-lactam substrates, except for cefepime and carbapenems, and are not inhibited by β-lactamase inhibitors, while MBLs are able to hydrolyze all clinical β-lactam substrates, with the exception of aztreonam [2]. Most BSBL-producing *Enterobacteriaceae* are also resistant to other commonly used antimicrobials, such as fluoroquinolones, aminoglycosides, and potentiated sulfonamides. In general, multidrug resistance (MDR) is defined as simultaneous resistance to antimicrobials of at least three different classes. Infections with these MDR bacteria impose serious public health threats, as they are associated with therapy failure and increased mortality rates in humans and animals [3].

Genes encoding BSBLs (*bla*-genes) have been found on chromosomes and mobile genetic elements, such as plasmids, transposons, and insertion sequences. As they allow intra- and inter-bacterial DNA mobility, mobile genetic elements have favored the dissemination of BSBLs in microbiomes of humans, animals and the environment worldwide [2,3]. Homologous BSBL genes and plasmid types have been identified in *Enterobacteriaceae* isolates from humans, animals, food, and the environment, suggesting BSBL transfer between different eco-systems. Direct contact with carrier animals, as well as handling and consumption of contaminated meat have been described as risk factors for BSBL transfer to humans [4].

Few studies, however, have investigated the potential role of zoo animals as reservoirs of BSBLs. Due to the popularity of zoos and their ability to bring exotic animals in close proximity to humans, zoos might act as BSBL reservoirs for humans [5]. Fecal carriage of ESBL- and AmpC-producing *Enterobacteriaceae* has already been shown in zoo animals from Japan, China, and the Czech Republic, with prevalence rates varying from 11% up to 32% [5,6,7,8]. In the report of Bender and Shulman, zoonotic disease outbreaks in humans, for example with *E. coli* O157, were associated with animal contact in petting zoos, farms and zoological parks [9]. Apart from transfer to humans, zoos may also facilitate transfer of resistant bacteria and/or resistance genes to other animals and environment, as animals are often exchanged during breeding programs and due to reintroduction of zoo animals and/or their offspring into the wild [10]. Furthermore, the increased popularity of free ranging animal exhibits in zoos may further facilitate interaction between other animals, humans and the environment [11].

Identification of BSBL reservoirs and risk factors associated with BSBL inter-sectorial transfer will be important to control further selection and dissemination of BSBL-producing bacteria. However, not much is known on the presence of BSBLs in zoos in Western Europe. In this study, we isolated and characterized BSBL-producing *Enterobacteriaceae* obtained from zoo mammals at two Belgian zoos.

## 2. Materials and Methods

### 2.1. Belgian Zoos

Zoo 1 has around 8000 animals of 590 different species, including 247 fish, 138 avian, 77 mammalian, 62 reptilian, 45 invertebrate, and 21 amphibian species. The zoo occupies a 0.11 km^2^ site in an urban region located in the northern part of Belgium and has around 1 million visitors per year. Zoo 2 houses more than 7000 animals of 587 different species, including 234 avian, 116 mammalian, 104 fish, 92 reptilian, 25 invertebrate, and 16 amphibian species. The zoo occupies a 0.75 km^2^ site in a rural region located in the southwestern part of Belgium and has around 2 million visitors per year. In both zoos, antimicrobials are only administered curatively on an individual basis for a limited period of time and under guidance of the veterinary officer. 

### 2.2. Selective Isolation from Fecal Samples 

Fresh fecal droppings were collected from 38 various terrestrial mammals. From Zoo 1, individual samples (*n* = 6) were obtained from 1 Amur tiger, 1 Western lowland gorilla, 1 Amur leopard and 3 spectacled bears (A–C), while mixed samples (*n* = 7) were taken from enclosures occupied by two or more individuals of the same species, namely 2 Asian elephants, 2 Eastern lowland gorillas, 2 siamangs, 3 African lions, 9 chimpanzees, 2 jaguars, and 3 dromedaries. From Zoo 2, individual samples (*n* = 25) were obtained from 2 Asian elephants (A–B), 2 African elephant (A–B), 2 Bengal tigers (A–B), 1 giraffe, 1 giant panda, 1 black and white ruffed lemur, 1 ring-tailed lemur, 2 white rhinoceros (A–B), 1 spotted hyena, 2 hippopotamuses (A–B), 2 African lions (A–B), 1 Sumatran orangutan, 1 snow leopard, 1 koala, 1 Tasmanian devil, 1 giant anteater, 1 South American tapir, 1 cheetah, and 1 Alpine marmot. 

One hundred mg feces was weighed from each sample, homogenized in 10 mL Buffered peptone water (Sigma-Aldrich, Saint Louis, MI, USA) and incubated overnight at 37 °C. After incubation, a swab was taken from the homogenized sample, streaked on McConkey III agar plates (Sigma-Aldrich) supplemented with 8 µg/mL ceftiofur (Sigma-Aldrich) and incubated overnight at 37 °C. Thereafter, all colonies differing in morphology were selected per sample and purified on Columbia agar plates (Oxoid, Basingstoke, UK). The purified colonies were identified using MALDI-TOF MS (Bruker Daltonics, Bremen, Germany) as described previously [12]. 

### 2.3. Antimicrobial Susceptibility Testing

Antimicrobial susceptibility testing of each isolate was determined using the disk diffusion method according to the Clinical Laboratory Standards Institute (CLSI) standards (M02, 2018). *Escherichia coli* ATCC 25922 was included as internal quality control. Isolates were tested for resistance against β-lactams (ampicillin (10 μg), cefalexin (30 μg), cefquinome (30 μg), ceftiofur (30 μg), amoxicillin-clavulanic acid (20 + 10 μg), imipenem (10 μg), and cefoxitin (30 μg)) (Neo-Sensitabs; Rosco Diagnostica, Taastrup, Denmark) to confirm the presence of β-lactamase producers. In addition, susceptibility to aminoglycosides (amikacin (30 μg), gentamicin (10 μg), neomycin (120 μg), and streptomycin (10 μg)), amphenicols (florphenicol (30 μg)), tetracyclines (tetracycline (30 μg) and doxycycline (30 μg)), nitrofurans (nitrofurantoin (100 μg)), fluoroquinolones (enrofloxacin (10 μg)), trimethoprim (5 μg) and trimethoprim-sulfamethoxazole (1.25 + 23.75 μg)) (Neo-Sensitabs; Rosco Diagnostica) was determined. Clinical resistance was based on CLSI breakpoints (M100, 2019). Multiple drug resistance was defined as simultaneous resistance to antimicrobials of at least three different classes [13]. 

### 2.4. Molecular Mechanisms of Resistance

Isolates with a BSBL phenotype were examined by PCR (*n* = 35), followed by gel electrophoresis and DNA sequencing for the presence of *bla*-genes encoding CTX-M, SHV, TEM and CMY β-lactamases, as previously described [14]. The obtained nucleotide sequences were compared with those previously described for *bla*-genes using BLAST with default parameters.

For all *E. coli* isolates showing presence of *bla*-gene(s) (*n* = 25), whole-genome sequencing (WGS) was performed to determine presence of a clonal expansion of the isolate(s) in or between both zoos. In addition, *E. marmotae* was included to investigate its phylogenetic relationship with the *E. coli* isolates. WGS was performed using the MinION long-read sequencing platform (Oxford Nanopore Technologies, Oxford, UK). In brief, isolates were cultivated overnight at 37 °C on Columbia agar plates, after which genomic DNA was isolated using the ZymoBiomics DNA MiniPrep Kit (Zymo Research, Irvine, CA, USA) according to the manufacturer’s instructions. Subsequently, high molecular weight DNA of each isolate was used for MinION library preparation using the Rapid Barcoding Kit (RBK0004, Oxford Nanopore Technologies). A MinION set-up was used for 48 h sequencing runs with a new FLO-MIN106D R9.4.1 flow cell. Quality of the isolation and sequencing procedure was verified by a positive control strain (*E. coli* ATCC 25922) and blank as negative control, included in each separate sequencing run.

Raw sequence read output files were base called using the high accuracy model of Guppy Basecaller GPU (v3.3.0, ONT), followed by demultiplexing of barcoded samples with qcat (v1.1.0, ONT). Subsequently, basecalled reads were filtered and trimmed using Nanofilt (v2.5.0) with a Q > 7 threshold [15]. Quality filtered reads were used as input for Flye (v2.6; [16]) de novo genome assembly, followed by read mapping against the generated draft assemblies with Graphmap (v0.5.2; [17]). Accuracy increased by polishing de novo assembled genomes using Racon GPU (v1.4.0, Clara Genomics, NVIDIA) and Medaka GPU (v0.10.1, ONT, Oxford, United Kingdom). Final taxonomic classification of polished assemblies was done using Kraken2 [18]. Computational speed of the bioinformatics pipeline increased by the integration of Geforce RTX 2080 Ti (GPU)-based computations where compatible with software. 

The resistome of all *Escherichia* spp. isolates was analyzed using the online software tools CARD [19] and Resfinder [20], after which the results were compared with the obtained antimicrobial susceptibility patterns. Antimicrobial resistance (AMR) gene clusters were defined as resistance genes present on the same contig within an isolate. Presence of plasmids was verified using PlasmidFinder in Abricate (v1.0.1; [21] and https://github.com/tseemann/abricate, accessed: 12 April 2021). In addition, the online software tool mlplasmids was used to predict whether the contigs containing AMR gene clusters were plasmid- or chromosome-derived, using *E. coli* as species model and 1000 bp as minimum sequence length [22]. Next, the online software tool ISfinder was used with default settings to detect presence of transposable elements in close proximity to the AMR gene clusters in the specific contigs [23], while ICEberg was used with default settings to detect presence of integrative and conjugative elements (ICEs) or integrative and mobilizable elements (IMEs) [24]. 

### 2.5. Phylogenetic Analysis and Strain Typing

All genomes were subjected to gene finding and automatic annotation using rapid prokaryotic genome annotation (Prokka) v1.14.5 [25]. Thereafter, pangenomes were created using rapid large-scale prokaryote pan genome analysis (Roary) v3.11.2 [26]. In brief, the annotated proteins from all isolates were used for a BLASTP all-versus-all sequence similarity search. From the BLASTP output, groups of orthologous proteins were predicted using the Orthagogue and MCL software [27]. Orthologous groups with exactly one representative protein from each of the input strains were considered to be part of the *Escherichia* spp. core genome. This obtained core genome alignment was then used for phylogenetic tree construction using randomized accelerated maximum likelihood (RAxML) v8.2.12 [28] by applying the -f a, -p 12345, -x 12345, -# 100, -m GTRGAMMA parameters and visualized using interactive tree of life (iTOL) (http://itol.embl.de/, accessed: 28 August 2020). 

To determinate clonal dissemination of the *Escherichia* spp. isolates, single nucleotide polymorphisms (SNP)-based mapping analysis was performed using the CSI Phylogeny 1.4 tool with default parameters [29] and with reference genome *E. coli* K-12 MG1655 (NC_000913.3) determined by the RefSeq NCBI Genome Database. The constructed tree was visualized using iTOL. In addition, multilocus sequence types were determined using the Pasteur (http://bigsdb.pasteur.fr, accessed: 28 August 2020) and Warwick [30] institute schemes, as well as phylogroups [31], serotypes [32], FimH and FumC types [33] and virotypes [34].

For Fourier-transform infrared spectroscopy, bacterial isolates were cultured on Mueller-Hinton for 22 ± 1 h at 35 ± 2 °C. A loopful of bacterial cells was suspended in 1.5 mL suspension vials with inert metal cylinders with 50 μL of 70% ethanol and vigorously vortexed after which 50 μL of HPLC water was added. After homogenization, 15 μL of each suspension were inoculated in triplicate on a silicon plate (Bruker Daltonics, Bremen, Germany). Two internal standards (IRTS1 and IRTS2) were spotted using a 12 µL volume according to the manufacturer’s guidelines after which the plates were dried for approximately 30 min. Spectra were acquired and processed by OPUS v7.5 and IR Biotyper software (Bruker Daltonics). Data from the area of polysaccharides (1300–800 cm^−1^) were vector normalized, and the second derivative was used to amplify differences between isolates. Hierarchical cluster analysis was done using the Euclidean average—mean spectra algorithm as available in the IR Biotyper software. These experiments were 3 times independently repeated.

## 3. Results

### 3.1. Bacterial Isolates and Antimicrobial Susceptibility

Thirty-five putatively ceftiofur-resistant isolates were obtained from fecal samples of 20 zoo mammals, while fecal samples of the other 18 zoo mammals were culture-negative. In more detail, 7/13 zoo mammals were found positive from Zoo 1 and 13/25 from Zoo 2. One isolate was selected from each fecal sample of 10 zoo mammals, whereas multiple morphologically different isolates were selected from the other 10 mammals, namely: 2 isolates from each fecal sample of 6 mammals, 3 isolates from each fecal sample of 3 mammals and 4 isolates from one mammal (Table 1). Using MALDI-TOF MS, most of the isolates were identified as *Enterobacteriaceae* (80.0%, 28/35), namely *E. coli* (89.2%, 25/28), *Escherichia marmotae* (3.6%, 1/28), *Klebsiella pneumoniae* (3.6%, 1/28) and *Citrobacter freundii* (3.6%, 1/28). Six isolates were identified as *Pseudomonas* sp. (17.1%, 6/35) and one isolate as *Achromobacter spanius* (2.9%, 1/35) (Table 1). All isolates showed an identification score value ≥ 2.0. 

The disk diffusion diameter results of *E. coli* ATCC 25922 fell within the acceptable quality ranges defined by CLSI (M100, 2019). Phenotypic resistance to β-lactam and non-β-lactam antimicrobials of the isolates is shown in Table 2. Potential ESBL and AmpC producers were identified in 84% and 16% of the *E. coli* isolates (21/25 and 4/25, resp.). Resistance to non-β-lactam antimicrobials was detected in 96% of the *E. coli* isolates (24/25), of which 64% showed multidrug resistance (16/25). Resistance to trimethoprim-sulfamethoxazole was most frequently detected (80%, 20/25), followed by tetracycline (64%, 16/25), doxycycline (60%, 15/25), streptomycin (40%, 10/25), trimethoprim (32%, 8/25), enrofloxacin (16%, 4/25) and gentamicin (12%, 3/25) resistance (Table 2). *E. marmotae* was identified as a potential ESBL producer, showing additional resistance against trimethoprim-sulfamethoxazole and trimethoprim. 

*K. pneumoniae* and *C. freundii* were identified as potential ESBL producers, showing additional resistance against enrofloxacin and against streptomycin and doxycycline, respectively. Two of the 6 *Pseudomonas* sp. isolates showed resistance to enrofloxacin, while *A. spanius* showed resistance to trimethoprim-sulfamethoxazole and trimethoprim (Table 2). 

### 3.2. Molecular Mechanisms of Resistance

By using PCR, all *E. coli* isolates showed presence of *bla*-gene(s), namely: 68% isolates carried the *blaCTX-M*-type gene (17/25), 28% isolates the *blaTEM* gene (7/25) and one isolate showed presence of both the *blaCTX-M*-type gene and the *blaTEM* gene. Sequence analysis revealed the following *bla*-gene types: CTX-M-1 (*n* = 17) and TEM-1 (*n* = 8) (Table 1). No *blaCMY* genes were found, despite presence of phenotypic AmpC producers. *E. marmotae* showed presence of the *blaCTX-M-type* gene, after which sequence analysis revealed it to be CTX-M-1. 

*K. pneumoniae* showed presence of both the *blaCTX-M*-type gene and the *blaSHV* gene, whereas *C. freundii* carried the *blaCMY* gene. Sequence analysis revealed it to be CTX-M-15, SHV-32 and CMY-124 respectively (Table 1). None of the *Pseudomonas* sp. isolates carried *bla*-gene(s), nor did *A. spanius*.

### 3.3. WGS and Resistome

For all *E. coli* isolates showing presence of *bla*-gene(s) (*n* = 25), WGS was performed to determine presence of a clonal expansion of isolate(s) in or between both zoos. In addition, *E. marmotae* was included to investigate its phylogenetic relationship with the *E. coli* isolates. The genome sizes ranged from 4.98 to 5.40 Mb and showed the following characteristics: 50.4 to 50.9% GC; 7016 to 8808 coding sequences; 661 to 949 hypothetical proteins and 107 to 115 RNAs.

Similar results were obtained when analyzing the resistome of the isolates using CARD and Resfinder (Additional files 1 and 2). Although the presence of *bla*-genes detected by PCR was confirmed, additional *bla*-genes were found, namely: *ampC* (all isolates, except for isolate µ (*E. marmotae*)), *DHA-1* (isolates E1-4), *CTX-M-3* (isolates T1-3) and *CTX-M-61* (isolate γ2). In general, the observed phenotypic resistance towards tetracyclines, fluoroquinolones, aminoglycosides, trimethoprim and/or sulfonamides could be linked with the presence of AMR genes (Table 3). Furthermore, other AMR genes, such as multidrug efflux pumps, were detected in all *Escherichia* spp. isolates (Additional files 1 and 2). 

Most of the AMR genes were located on plasmids, except for *CTX-M-1* of isolates L1-3 and the *CTX-M-3*, *mphA*, and AAC*(3)-IIc* cluster of isolates T1-3, which were located on chromosomes (Table 3). AMR gene clusters were observed in 18/26 *Escherichia* spp. isolates, and most often between *bla*-genes and genes encoding resistance to sulfonamides and/or tetracyclines. Furthermore, the AMR gene clusters could be linked with transposases and/or putative predicted IMEs or ICEs (Table 3). Additional information on plasmid replicon typing and identification can be found in Additional file 3. 

### 3.4. Phylogenetic Analysis and Strain Typing

As shown in Table 4, isolates showing identical multilocus sequence types also shared the same phylogroup, serotype, FimH, and FumC type and virotype. This shared homology was observed for all isolates obtained from the same animal, except for κ1 and κ2 obtained from the South American tapir. Strain type homology was also observed between isolates B, I1-2, and K1-2 obtained from the Amur tiger, Amur leopard and spectacled bear_C at Zoo 1, respectively, and between isolates V1-2 and X1-3 obtained from the spotted hyena and African lion_A at Zoo 2, respectively. In general, phylogroup B1 and virotype B were most frequently identified (i.e., 13/26 and 20/26, resp.).

**Figure 1:**
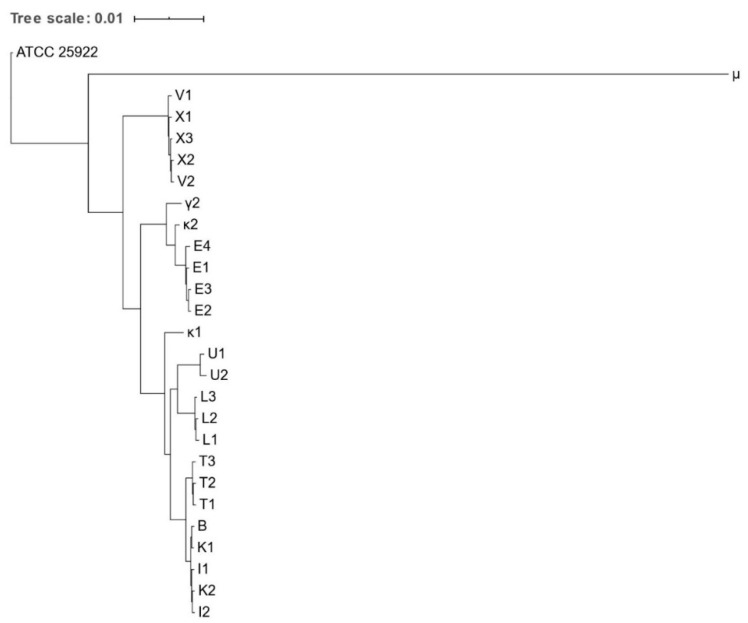
Phylogenetic tree based on the concatenated core gene alignment of *Escherichia* spp. isolates obtained from various zoo mammals. The scale-bar represents 1% differences in nucleotide sequences. All isolates were identified as *E. coli*, except for μ, which was identified as *E. marmotae*.

**Figure 2:**
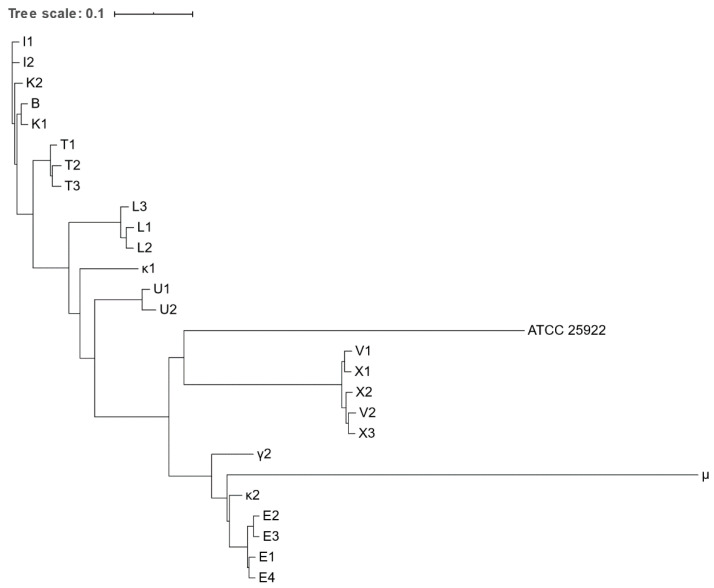
Phylogenetic tree based on the concatenated alignment of the high-quality SNPs alignment of *Escherichia* spp. isolates obtained from various zoo mammals. The scale-bar represents 1% differences in nucleotide sequences. All isolates were identified as *E. coli*, except for μ, which was identified as *E. marmotae*.

**Figure 3:**
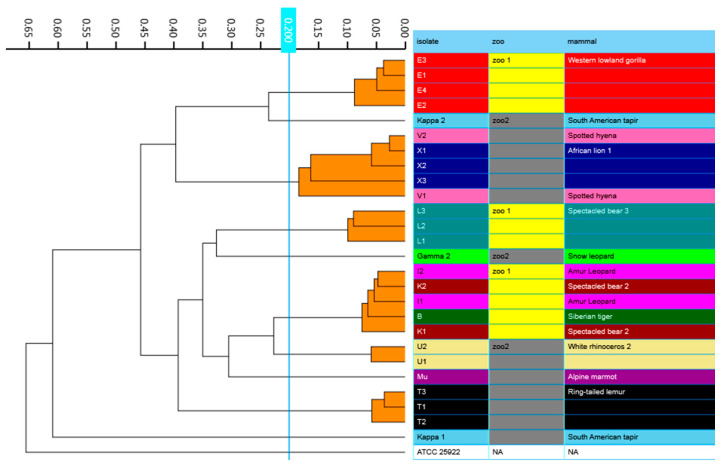
IR Biotyper generated dendrogram showing clustering of *Escherichia* spp. isolates obtained from various zoo mammals. Isolates that belong to the same cluster, using a cut-off of 0.200, are indicated with orange branches. All isolates were identified as *E. coli*, except for μ, which was identified as *E. marmotae*.

Phylogenetic trees based on core genome and SNPs alignment as well as the IR Biotyper dendrogram confirmed clustering of all isolates obtained from the same animal species, except for κ1 and κ2 (Figure 1, Figure 2 and Figure 3). Clustering of isolates B, I1-2, and K1-2 and of isolates V1-2 and X1-3 was demonstrated as well. 

## 4. Discussion

In this study, around 37% of the sampled zoo mammals showed fecal carriage of BSBL-producing *Enterobacteriaceae* (i.e., 14/38), thereby exceeding previously reported rates of 11% up to 32% for zoo animals in Japan, China, and the Czech Republic [5,6,7,8]. These discrepancies might be explained by the relatively low number of sampled animals in this study (*n* = 38), sampling of different animal species, geographical differences and/or differences in zoo management. It is also possible that the prevalence of BSBL producers in zoo animals is currently being underestimated due to the limited number of studies. In comparison, presence of BSBLs in food-producing animals has been intensively studied, with reported prevalence rates of up to 89% [1]. Our results might also indicate that the prevalence of BSBL-producing bacteria in zoo animals has been increasing, similar to described for humans, pets and food-producing animals [3]. It would be interesting to perform more studies to determine the real burden of BSBLs in zoo animals. 

In line with previous studies, most BSBL-producing isolates were identified as *E. coli*, with a predominance of the ESBL phenotype and only few AmpC producers. Furthermore, CTX-M-1 was the predominant ESBL in the fecal flora of zoo animals, similar to the situation in pets, food-producing animals and wild birds [1,5]. Although the pathogenic significance of *E. coli* strains carrying this enzyme is unclear, a higher percentage of CTX-M-1 producers has been reported in sick companion- and food-producing animals compared to healthy ones (i.e., 30% vs. 23%, resp.) [1]. Nevertheless, sick animals are often treated with first generation cefalosporins, penicillins and/or amoxicillin, which may have contributed to an increased prevalence of CTX-M-1. Both in our study and the one of Dabiosova et al., all zoo animals showing presence of CTX-M-1 producers were clinically healthy [5], although we were unable to obtain information of previous antimicrobial treatments. 

Additional clustering was shown between five *E. coli* isolates obtained from an Amur tiger, an Amur leopard and a spectacled bear from Zoo 1, and between five *E. coli* isolates obtained from a spotted hyena and an African lion from Zoo 2. These findings indicate that a clonal expansion of a *E. coli* strain occurred in both zoos. Transmission of both clones may have occurred through multiple pathways, of which shared meat diets may be an important one. Indeed, ESBL- and AmpC-producing *E. coli* strains are frequently found in meat, with prevalence rates of up to 100% [35]. This might also provide an explanation why ESBL- and AmpC-producing *E. coli* strains were slightly more frequently isolated from carnivorous zoo mammals (7/11) than herbivorous ones (4/11). Nevertheless, other pathways may have contributed to the clonal expansion, as the spectacled bear’s diet differed from those of the Amur tiger and Amur leopard from Zoo 1. For example, both *E. coli* strains may have been transferred via mechanical vectors, as these animals are housed next to each other and have the same animal caretakers. 

For the first time, an ESBL producing and MDR *E. marmotae* was isolated from an Alpine marmot (*Marmota marmotae*). So far, in only one other study this bacterium was isolated from the feces of wild Himalayan marmots (*M. himalayana*) [36]. The phylogenetic trees based on (i) core genome and (ii) SNPs alignment as well as (iii) FTIS confirmed a separate clustering of the *E. marmotae* isolate from the *E. coli* isolates. Although the presence of virulence associated genes and cell invasion experiments in vitro already suggested that *E. marmotae* is likely to be an invasive pathogen [36], further studies will be required to demonstrate its pathogenicity and zoonotic potential. 

Besides *Escherichia* species, *K. pneumoniae*, *Pseudomonas* sp. and *C. freundii* were isolated from 21% of the sampled zoo mammals (8/38). All three species are well described ubiquitous and opportunistic animal and human pathogens, with reported cases of zoonotic and/or anthropozoonotic transmission [37,38]. For the first time, however, the *blaCMY-124* gene was shown to be present in *C. freundii* isolated from a Tasmanian devil, as well as the combination of CTX-M-15 and SHV-32 in *K. pneumoniae* isolated from an African lion. Further epidemiological studies in zoo animals should be performed to estimate the real burden of these *bla*-gene clusters.

Most of the obtained isolates were MDR and this mainly against (potentiated) sulfonamides and tetracyclines, as already shown by others [8]. Although the underlying mechanism of multidrug resistance development in this study is unclear, previous antimicrobial treatments may have played a role. Indeed, Ishihara et al. linked the use of amoxicillin as first-line therapy in zoo animals with phenotypic resistance to ampicillin. Resistance to kanamycin, gentamicin, trimethoprim and tetracycline was also significantly higher in animals treated with ampicillin, indicating co-localization of antimicrobial resistance genes on mobile genetic elements [7]. Indeed, in this study, almost all AMR genes were located on plasmids containing transposons. Apart from treatment, transfer of resistant bacteria and/or horizontal transfer of resistance genes through feed, humans and surroundings may also contribute to multidrug resistance development [10]. Since we were unable to obtain in-depth information on previous treatments, food source origin, etc., it would be interesting to investigate this in a future survey. Identifying risk factors will be necessary to control further selection and dissemination of MDR bacteria in zoo mammals.

The presence of MDR bacteria in zoo mammals may impose health risks for visitors and animal caretakers of Belgian zoos. As already described for farm animals [1], zoo animals may serve as a reservoir and disseminator of zoonotic pathogens and antimicrobial resistance for humans, other animals, and the environment. To minimize spread and dissemination, it can be advised to apply additional hygienic measures in zoos, such as eating and drinking restrictions and provision of hand-sanitizing facilities, especially in petting areas, as well as frequent surface disinfection and removal of animal feces [39]. Further research, however, remains necessary to investigate if these pathogens and/or multidrug resistance effectively transfer from zoo mammals to humans and whether this transfer is related to health risks. In that light, special attention should be made for animal caretakers, as they have more intense and frequent contact with infected animals. 

## 5. Conclusions

MDR BSBLs were shown to be present in the fecal microbiota of zoo mammals in two zoos in Belgium. Interestingly, a clonal expansion of a *E. coli* strain may have occurred in both zoos between different animal species, most likely through shared meat diets and/or localization of animal enclosures next to each other. Further research is necessary to investigate if these MDR BSBLs effectively transfer to humans and whether this transfer poses health risks.

## Figures and Tables

**Table 1:** Identification of 35 ceftiofur-resistant isolates obtained from fecal samples of zoo mammals and overview of detected *bla*-genes in these isolates.

Zoo	Zoo Mammal	Isolate	Identification	*bla*-Genes
1	Amur tiger	B	*E. coli*	CTX-M-1
	Western lowland gorilla	E1	*E. coli*	TEM-1
		E2	*E. coli*	TEM-1
		E3	*E. coli*	TEM-1
		E4	*E. coli*	TEM-1
	Amur leopard	I1	*E. coli*	CTX-M-1
		I2	*E. coli*	CTX-M-1
	Spectacled bear_B	K1	*E. coli*	CTX-M-1
		K2	*E. coli*	CTX-M-1
	Spectacled bear_C	L1	*E. coli*	CTX-M-1
		L2	*E. coli*	CTX-M-1
		L3	*E. coli*	CTX-M-1
2	Ring-tailed lemur	T1	*E. coli*	TEM-1
		T2	*E. coli*	TEM-1
		T3	*E. coli*	TEM-1
	White rhinoceros_B	U1	*E. coli*	CTX-M-1
		U2	*E. coli*	CTX-M-1
	Spotted hyena	V1	*E. coli*	CTX-M-1
		V2	*E. coli*	CTX-M-1
	African lion_A	X1	*E. coli*	CTX-M-1
		X2	*E. coli*	CTX-M-1
		X3	*E. coli*	CTX-M-1
	Snow leopard	γ2	*E. coli*	TEM-1; CTX-M-1
	South American tapir	κ1	*E. coli*	CTX-M-1
		κ2	*E. coli*	CTX-M-1
	Alpine marmot	μ	*E. marmotae*	CTX-M-1
	African lion_B	δ	*Klebsiella pneumoniae*	CTX-M-15; SHV-32
	Tasmanian devil	θ	*Citrobacter freundii*	CMY-124
1	Dromedaries (*n* = 3)	M	*Pseudomonas* sp.	/
2	Asian elephant_A	N	*Pseudomonas* sp.	/
	Asian elephant_B	Z1	*Pseudomonas* sp.	/
		Z2	*Pseudomonas* sp.	/
	Sumatran orangutan	β	*Pseudomonas* sp.	/
	Hippopotamus_A	η	*Pseudomonas* sp.	/
1	Eastern lowland gorillas (*n* = 2)	F	*Achromobacter spanius*	/

Species identification was performed using MALDI-TOF; *bla*-genes were detected using PCR; / = no *bla*-genes were detected; all isolates were obtained from individual animals, except for M and F which were isolated from a mixed sample of three dromedaries and two Eastern lowland gorillas, respectively.

**Table 2:** Phenotypic resistance to β-lactam and non-β-lactam antimicrobials of 35 ceftiofur-resistant isolates obtained from zoo mammals.

Isolate		AMP	AMC	CFO	CFLEX	CFQUI	CFTIO	IMI	GEN	ENROF	TRIM	SxT	NEOMY	DOX	TET	STR	FFC	AMI	NI
*E. coli*	B	R	S	S	R	R	R	S	S	S	S	R	S	R	R	I	S	S	S
	E1	R	R	R	R	S	R	S	S	S	R	R	I	R	R	R	S	S	S
	E2	R	R	R	R	S	R	S	S	S	R	R	S	R	R	R	S	S	S
	E3	R	R	R	R	S	R	S	S	I	R	R	S	R	R	R	S	S	S
	E4	R	R	R	R	S	R	S	S	I	R	R	S	R	R	R	S	I	S
	I1	R	S	S	R	R	R	S	S	S	S	R	I	R	R	I	S	S	S
	I2	R	S	S	R	R	R	S	S	R	S	S	I	R	R	R	S	I	S
	K1	R	S	S	R	R	R	S	S	S	S	R	S	R	R	I	S	S	S
	K2	R	S	S	R	R	R	S	S	S	S	R	I	R	R	I	S	S	S
	L1	R	S	S	R	R	R	S	S	S	S	R	S	R	R	I	S	S	S
	L2	R	S	S	R	R	R	S	S	S	S	R	S	R	R	I	S	S	S
	L3	R	S	S	R	R	R	S	S	S	S	R	S	R	R	I	S	S	S
	T1	R	S	S	R	R	R	S	R	R	R	R	R	R	R	R	I	S	S
	T2	R	S	S	R	R	R	S	R	R	R	R	R	R	R	R	S	S	S
	T3	R	S	S	R	R	R	S	R	R	R	R	R	R	R	R	S	S	S
	U1	R	S	S	R	R	R	S	S	S	S	S	I	S	S	R	S	S	S
	U2	R	S	S	R	R	R	S	S	S	S	S	S	S	S	R	S	S	S
	V1	R	S	S	R	R	R	S	S	S	S	R	S	S	S	I	S	S	S
	V2	R	S	S	R	R	R	S	S	S	S	R	S	S	S	I	S	S	S
	X1	R	S	S	R	R	R	S	S	S	S	R	S	S	S	S	S	S	S
	X2	R	S	S	R	R	R	S	S	S	S	R	I	S	S	I	S	S	S
	X3	R	S	S	R	R	R	S	S	S	S	R	S	S	S	S	S	S	S
	γ2	R	S	S	R	R	R	S	S	S	S	R	S	S	R	S	S	S	S
	κ1	R	S	S	R	R	R	S	S	S	R	S	I	S	S	S	S	S	S
	κ2	R	S	S	R	R	R	S	S	S	S	S	S	S	S	S	S	S	S
*E. marmotae*	μ	R	S	S	R	R	R	S	S	S	R	R	I	S	S	S	S	S	S
*E. coli* ATCC 25922		S	S	S	S	S	S	S	S	S	S	S	S	S	S	I	S	S	S
*Klebsiella pneumoniae*	δ	R *	S	S	R	R	R	S	S	R	S	S	I	S	S	S	S	S	S
*Citrobacter freundii*	θ	R *	R *	R	R *	S	R	S	S	S	S	S	I	R	S	R	S	S	S
*Pseudomonas* sp.	M	R *	R *	R *	R *	S	R *	S	S	S	R *	R *	R *	R *	R *	R *	R *	S	R *
	N	R *	R *	R *	R *	S	R *	S	S	S	R *	R *	R *	R *	R *	R *	R *	S	R *
	β	R *	R *	R *	R *	S	R *	S	S	R	R *	R *	R *	R *	R *	R *	R *	S	R *
	Z1	R *	R *	R *	R *	S	R *	S	S	I	R *	R *	R *	R *	R *	R *	R *	S	R *
	Z2	R *	R *	R *	R *	S	R *	S	S	I	R *	R *	R *	R *	R *	R *	R *	S	R *
	η	R *	R *	R *	R *	I	R *	S	S	R	R *	R *	R *	R *	R *	R *	R *	S	R *
*Achromobacter spanius*	F	R *	S	R *	R *	S	R *	S	S	S	R	R	S	S	S	R *	S	S	S

* = intrinsic resistance; R = resistant, I = intermediate, S = sensitive, AMP = ampicillin (10 μg), AMC = amoxicillin-clavulanic acid (20 + 10 μg), CFO = cefoxitin (30 μg), CFLEX = cefalexin (30 μg), CFQUI = cefquinome (30 μg), CFTIO = ceftiofur (30 μg), IMI = imipenem (10 μg), GEN = gentamicin (10 μg), ENROF = enrofloxacin (10 μg), TRIM = trimethoprim (5 μg), SxT = trimethoprim-sulfamethoxazole (1.25 + 23.75 μg), NEOMY = neomycin (120 μg), DOX = doxycycline (30 μg), TET = tetracycline (30 μg), STR = streptomycin (10 μg), FFC = florphenicol (30 μg), AMI = amikacin (30 μg), NI = nitrofurantoin (100 μg).

**Table 3:** Overview, clustering, and localization of AMR genes detected in *Escherichia* spp. isolates obtained from zoo mammals.

Isolate	Potential Important AMR Genes	Phenotypic Resistance	Clustered AMR Genes †	Linked Transposases or IME ‡	Predicted Contig Origin §
B	β-lactams: *ampC1, CTX-M-1*	Yes	*CTX-M-1, tet(C) and sul 2*	IS1294, ISEcp1, IS186B, and IS5075	Plasmid
	Tetracyclines: *tet(A), tet(C)*	Yes			
	Sulphonamides: *sul2*	Yes			
E1-4	β-lactams: *ampC1, TEM-1, DHA-1*	Yes	*TEM-1, tet(B), tetR, sul2, APH(3″)-Ib and APH(6)-ld*	Tn2 and IS5	Plasmid
	Tetracyclines: *tet(B), tetR*	Yes			
	Sulphonamides: *sul1, sul2*	Yes			
	Trimethoprim: *dfrA8, dfrA17*	Yes			
	Aminoglycosides: *APH(6)-Id, APH(3″)-Ib*	Yes	*QnrB4, sul1, mphA, and dfrA17*	IS186B, IS1R, and Tn2	Plasmid
	Macrolides: *mphA*	/			
	Fluoroquinolones: *QnrB4*	No			
I1-2	β-lactams: *ampC1, CTX-M-1*	Yes	*CTX-M-1, tet(C) and sul 2*	ISEcp1, IS1294, and IS186B	Plasmid
	Tetracyclines: *tet(C)*	Yes			
	Sulphonamides: *sul2*	Yes			
	Aminoglycosides: *efflux pumps*	Yes			
	Fluorquinolones: *efflux pumps*	Yes			
K1-2	β-lactams: *ampC1, ampC, CTX-M-1*	Yes	*CTX-M-1, tet(A), tet(D) and sul2*	ISEcp1, IS1294, and IS186B	Plasmid
	Tetracyclines: *tet(A), tet(D)*	Yes			
	Sulphonamides: *sul2*	Yes			
L1-3	β-lactams: *ampC1, CTX-M-1*	Yes	*CTX-M-1*	ISEcp1; putative ICE with T4SS	Chromosome
				
	Tetracyclines: *tet(A)*	Yes			
	Sulphonamides: *sul2*	Yes	*tet(A) and sul2*	IS1294 and IS5075	Plasmid
T1-3	β-lactams: *ampC1, CTX-M-3, TEM-1*	Yes	*CTX-M-3, mphA, and AAC(3)-IIc*	Tn2; Putative IME	Chromosome
	Tetracyclines: *tet(B), tetR*	Yes			
	Sulphonamides: *sul2*	Yes			
	Trimethoprim: *dfrA17*	Yes			
	Aminoglycosides: *APH(3′)-Ia, APH(6)-Id, APH(3″)-Ib, aadA5, AAC(3)-IIc*	Yes	*TEM-1, tet(B), dfrA17, sul2, APH(3′)-Ia, APH(6)-Id, APH(3″)-Ib and aadA5*	Tn2, ISEc8, IS3411, IS1R, and IS2	Plasmid
	Macrolides: *mphA*	/			
	Fluoroquinolones: *gyrA, parC*	Yes			
U1-2	β-lactams: *ampC1, ampC, CTX-M-1*	Yes	*CTX-M-1*	ISEcp1 and IS1294	Plasmid
	Aminoglycosides: *efflux pumps*	Yes			
	Fluoroquinolones: *gyrA*	No			
V1-2	β-lactams: *ampC1, CTX-M-1*	Yes	*CTX-M-1, sul2*	IS5075, IS1294, and ISEcp1	Plasmid
	Sulphonamides: *sul2*	Yes			
X1-3	β-lactams: *ampC, CTX-M-1*	Yes	*CTX-M-1, sul2*	IS5075, IS1294, and ISEcp1	Plasmid
	Sulphonamides: *sul2*	Yes			
γ2	β-lactams: *ampC1, ampC, CTX-M-61, TEM-1*	Yes	*CTX-M-61, TEM-1, sul2*	Tn2, ISEcp1, IS2, IS1294, and IS5075	Plasmid
	Tetracyclines: *efflux pumps*	Yes			
	Sulphonamides: *sul2*	Yes			
κ1	β-lactams: *ampC1, CTX-M-1*	Yes	*CTX-M-1*	ISEcp1 and IS1294	Plasmid
	Trimethoprim: *dfrA5*	Yes			
Κ2	β-lactams: *ampC1, ampC, CTX-M-1*	Yes	*CXT-M-1*	ISEcp1, IS1294	Plasmid
μ	β-lactams: *CTX-M-1*	Yes	*CTX-M-1*	ISEcp1 and IS1294	Plasmid
	Sulphonamides: *sul2*	Yes			
	Trimethoprim: *dfrA17*	Yes	*sul2, dfrA17, aadA5*	IS5075 and TnEc3	Plasmid
	Aminoglycosides: *aadA5*	No			
ATCC 25922	/	/	/	/	/

All isolates were identified as *E. coli*, except for μ, which was identified as *E. marmotae*. AMR = antimicrobial resistance; IME = integrative mobilizable elements; ICE = integrative and conjugative element; † = AMR genes present on the same contig; ‡ = presence of transposases and IME were determined by ISfinder and ICEberg, respectively; § = localization of AMR genes on plasmid or chromosome was determined by mlplasmids.

**Table 4:** Overview of the multilocus sequence type, phylogroup, serotype, FimH, and FumC type and virotype of the *Escherichia* spp. isolates obtained from zoo mammals.

Isolate	Pasteur ST	Warwick ST	Phylogroup	Serotype	CHTyper	Virotype
B	294	162 *	B1	O8:H28	fumC65 fimH32	B
E1	2	10929 *	C	O16:H48	fumC11 fimH475	/
E2	2	10929 *	C	O16:H48	fumC11 fimH475	/
E3	2	10929 *	C	O16:H48	fumC11 fimH475	/
E4	2	10929 *	C	O16:H48	fumC11 fimH475	/
I1	294	162 *	B1	O8:H28	fumC65 fimH32	B
I2	294	162 *	B1	O8:H28	fumC65 fimH32	B
K1	294	162 *	B1	O8:H28	fumC65 fimH32	B
K2	294	162 *	B1	O8:H28	fumC65 fimH32	B
L1	529	Unknown ST; Nearest match: 180, 675	B1	O22:H16	fumC23 fimH32	B
L2	529	Unknown ST; Nearest match: 180, 675	B1	O22:H16	fumC23 fimH32	B
L3	529	Unknown ST; Nearest match: 180, 675	B1	O22:H16	fumC23 fimH32	B
T1	355	162 *	B1	O55:H10	fumC65 fimH32	B
T2	355	162 *	B1	O55:H10	fumC65 fimH32	B
T3	355	162 *	B1	O55:H10	fumC65 fimH32	B
U1	Unknown ST; Nearest match: 325	1844	B1	O8:H49	fumC29 fimH38	B
U2	Unknown ST; Nearest match: 325	1844	B1	O8:H49	fumC29 fimH38	B
V1	42	57 *	E	O140:H25	fumC31 fimH27	B
V2	42	57 *	E	O140:H25	fumC31 fimH27	B
X1	42	57 *	E	O140:H25	fumC31 fimH27	B
X2	42	57 *	E	O140:H25	fumC31 fimH27	B
X3	42	57 *	E	O140:H25	fumC31 fimH27	B
γ2	843	1564 *	A	0-:H21	fumC252 fimH /	B
κ1	24	Unknown ST; Nearest match: 58, 223	Unknown	O8:H25	fumC4 fimH32	B
κ2	165	10 *	C	O16:H12	fumC11 fimH24	/
μ	Unknown ST; Nearest match: 606	8370 *	F	O13:H56	fumC48 fimH150/160	/
ATCC 25922	52	Unknown ST; Nearest match: 73, 5999	B2	O6:H1	fumC24 fimH30	/

All isolates were identified as *E. coli*, except for μ, which was identified as *E. marmotae*. ST = sequence type; * = three up to five alleles with an identity between 99.1% and 99.8% were found; / = no match was found.

## Data Availability

The sequences of all *Escherichia* spp. isolates have been deposited in DDBJ/ENA/GenBank under bioproject (PRJNA662678) with accession numbers JACWBW000000000-JACWBF000000000.

## Supplementary Materials

The following are available online at https://www.mdpi.com/article/10.3390/microorganisms9040834/s1, Table S1: Additional File 1.xlsx, Table S2: Additional File 2.xlsx, TableS3: Additional File 3.xlsx
